# The Contribution of Diet Therapy and Probiotics in the Treatment of Sarcopenia Induced by Prolonged Immobilization Caused by the COVID-19 Pandemic

**DOI:** 10.3390/nu14214701

**Published:** 2022-11-07

**Authors:** Carmen Delia Nistor-Cseppento, Titus David Moga, Alexa Florina Bungau, Delia Mirela Tit, Nicoleta Negrut, Bianca Pasca, Calin Florin Bochis, Timea Claudia Ghitea, Anamaria Jurcau, Anamaria Lavinia Purza, Diana Uivarosan

**Affiliations:** 1 Department of Psycho-Neuroscience and Recovery, Faculty of Medicine and Pharmacy, University of Oradea, 410073 Oradea, Romania; 2 Department of Morphological Disciplines, Faculty of Medicine and Pharmacy, University of Oradea, 410073 Oradea, Romania; 3 Doctoral School of Biological and Biomedical Sciences, University of Oradea, 410087 Oradea, Romania; 4 Department of Pharmacy, Faculty of Medicine and Pharmacy, University of Oradea, 410028 Oradea, Romania; 5 Clinical Oro-Maxillo-Facial Surgery, Clinical Emergency Municipal Hospital Timisoara, 300062 Timișoara, Romania; 6 Department of Preclinical Disciplines, Faculty of Medicine and Pharmacy of Oradea, University of Oradea, 410073 Oradea, Romania

**Keywords:** sarcopenia, muscle mass index, diet therapy, probiotics, COVID-19, SARS-CoV-2, albumin (Alb), hemoglobin (Hb)

## Abstract

The prolonged immobilization associated with COVID-19 infection and the restrictions imposed by the pandemic have determined major changes in physical activity and eating habits, with a negative impact on physical performance. This study monitored non-pharmacological interventions (diet therapy and probiotics) in managing sarcopenia for patients with recent SARS-CoV-2 history (14 days). A prospective study was performed on 200 patients (between December 2020–December 2021), with SPPB score < 9, randomly divided into: Group K—DP (93 patients) with dietary therapy (protein 1.2–1.5 g/kg) and probiotics for two months; and Group K—non-DP (107 patients) without diet therapy and probiotics. All patients were included in a specific physical training program (40 min), three sessions per week. Skeletal muscle index (SMI), serum albumin, and hemoglobin were determined. The SMI was initially low for both groups without significant statistical differences (6.5 ± 0.52 kg/m^2^ for Group K—non-DP vs. 6.7 ± 0.57 Kg/m^2^ for Group K—DP, *p* = 0.135). After two months, significant difference between initial and final SMI values was determined for Group K—DP (6.92 ± 0.50 kg/m^2^ vs. 6.77 ± 0.56 kg/m^2^, *p* = 0.048). In Group K—DP, at end of study, were more patients with normal SMI (n = 32 → N = 70) values (*p* < 0.001) and fewer sarcopenia patients (*p* < 0.001). The initial serum albumin means values in the two groups (Group K—non-DP, 4.17 ± 1.04 g/dL, and Group K—DP, 3.95 ± 0.98 g/dL) were not statistically significantly different (*p* = 0.122). The hemoglobin level improved significantly following a hyper protein diet enriched with pro-biotics (*p* = 0.003). Diet therapy, consisting of increased protein intake and specific probiotics and specific physical therapy, demonstrated superiority in improving the functional status of patients with recent COVID-19 infection.

## 1. Introduction

**Figure 1:**
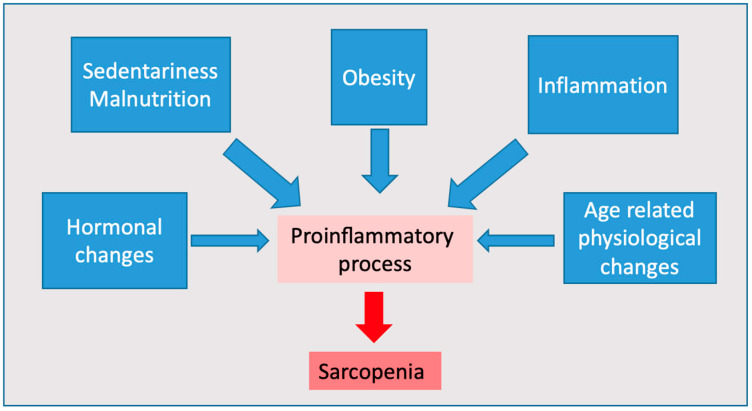
Causes contributing to the onset of sarcopenia.

Sarcopenia is defined as the progressive loss of muscle mass having an impact on the physical performance of individuals [1]. The literature describes two clinical forms of sarcopenia: primary and secondary. The primary form appears with aging, no other causes have been identified [1]. The secondary form appears when determining factors are present (comorbidities, inflammatory factors, sedentariness, immobility, and poor dietary intake) [2]. Physical performance (expressed through mobility), balance, coordination, and agility, correlates with muscle mass [3], the etiology of this disorder is multi-factorial. Aging, associated with chronic diseases, inadequate dietary intake, changes in muscle fibers (qualitative and quantitative), and hormonal deficiency are some of the causes involved in the pathophysiology of sarcopenia. Chronic inflammation plays a central role in its development; thus, it alters cellular protein metabolism favoring proteolysis, reducing synthesis, and thus accelerating muscle atrophy [4], as it is described in the Figure 1.

Obesity is correlated with increased incidence of sarcopenia [5]. Pro-inflammatory biomarkers of systemic inflammation (tumor necrosis factor (TNFα), Interleukin-6 (Ile-6), act synergistically in the development of sarcopenia, which activates the ubiquitin-proteasome system. The result is accelerated protein degradation and reduction in skeletal muscle mass. Among the hematological biomarkers identified as being correlated (negatively) with sarcopenia are albumin (Alb) and hemoglobin (Hb) [6]. Fibroblast growth factor (FGF21) is a molecule with a catabolic effect on muscle metabolism. Serum FGF21 level correlates with sarcopenia phenotype and is inversely proportional to skeletal muscle mass index [7].

Sarcopenia may occur as a result of physical inactivity [8]. Several investigations revealed that exercise boosts older persons’ muscular mass and strength [9,10]. Consequently, a direct correlation between physical activity and a lower incidence of sarcopenia was found. Resistance exercise, in particular, is typically thought to be the strongest protection against sarcopenia [11].

The COVID-19 outbreak that emerged in late 2019 has spread globally; on 11 March 2020, it was declared a pandemic. This has led to significant lifestyle changes, with isolation, decreased physical activity, changes in diet [12,13,14], and even the possibility of infection with clinical manifestations and various complications. Hospitalization or prolonged bed rest caused by SARS-CoV-2 infection reduces muscle and bone mass, with the emergence of a new syndrome called osteosarcopenia [15]. Moreover, in this context, the “catabolic crisis” [13] triggered by prolonged immobilization and isolation caused by COVID-19 infection is discussed. This may be responsible for the patient’s prognosis for discharge as well as for the patient’s functional status after discharge. The hyperinflammatory state caused by SARS-CoV-2 leads to altered mitochondrial function, autophagy, myofibril, and muscle degradation. Inflammatory status, reduced physical activity, obesity, and presence of diabetes mellitus interact with the mTORc1 pathway (involved in mitochondrial turnover) causing reduced muscle protein synthesis. In addition to these aspects, the gut microbiota is also affected, leading to a decreased appetite for food and exacerbating catabolism [16]. The resulting dysbiosis leads to increased gut permeability (more pronounced in the elderly). Inflammation initially occurs at the intestinal level, then at the systemic level [2]. The result is reduced anabolism and protein synthesis, together with insulin resistance [17].

This requires a multidisciplinary approach of patients with a history of COVID-19 infection, including a proper diet (hyper protein), adequate physical training, and psychological support. Leucine is an essential amino acid (AA) that plays a role in stimulating mTORC1 phosphorylation. It has been hypothesized that this amino acid stimulates appetite and decreases proteolysis [4]. The unhealthy diet associated with lack of activity and the inflammatory state caused by coronavirus infection trigger sarcopenic obesity. Attempts have been made to treat sarcopenia by leucine supplementation showing that leucine ingestion may have a favorable effect on muscle protein synthesis in the elderly. The results have not been sustained long term. During COVID-19 infection, alteration of the olfactory and gustatory epithelium [16], (SARS-CoV-2 occupies the sialic acid binding sites on the taste buds [18]) and the presence of neurasthenia and depression [19] can be the causes of deficiency in the intake of essential amino acids, vitamins, and other essential microelements [20]. Poor nutrition may be associated with poor oral hygiene or edentulousness. 

The literature refers to sarcopenic dysphagia, caused by sarcopenia of the skeletal and swallowing muscles, and presbyphagia (difficulty swallowing, characteristic of the elderly in tarry conditions) [21]. Fever and inflammation, characteristic for infection, increase caloric requirements. Certainly, inadequate caloric intake leads to the development of sarcopenia. Other aggravating factors are anorexia, diarrhea, and reduced assimilation. The type of protein ingested is also essential in the intestinal absorption of AA. Animal proteins are absorbed more efficiently than plant proteins (which may be deficient in essential AA, e.g., peas are deficient in methionine, which plays a vital role in muscle metabolism) [22]. The importance of treatment with probiotics that influence the intestinal microbiota is therefore emphasized, favoring the absorption of proteins (especially plant proteins) [23,24].

Our study aimed to monitor non-pharmacological interventions (diet therapy and probiotics) in managing sarcopenia in patients with a recent (14 days) history of SARS-CoV-2. To highlight the effect of therapeutic interventions on sarcopenia, skeletal muscle index (SMI), physical performance, along with paraclinical parameters (Alb, Hb) were evaluated. Moreover, this research complements other results published by our group [13] regarding the effects of prolonged immobilization caused by the COVID-19 pandemic. 

## 2. Materials and Methods

### 2.1. Study Design

A prospective study was conducted between December 2020 and December 2021 at the Medical Treatment and Rehabilitation Centre, Băile 1 Mai, Ceres Hotel, Bihor County, Romania, in collaboration with the private medical practice of Echo Laboratories, Oradea, Romania. Inclusion criteria that were taken into account for subjects’ selection were recent history of COVID-19 infection and low physical performance, and an SPPB score < 9. Exclusion criteria considered the history of malignancy, articular ankylosis, organ failure, and refusal of patients to participate in the study. The age of the subjects was not a selection criterion, because most of those who usually turn to the recovery service are over 60 years of age, the center being recognized as specifically serving retirees. Moreover, it must be mentioned that all patients were stabilized after COVID-19, without organ failure, and able to undergo recovery treatment.

**Figure 2:**
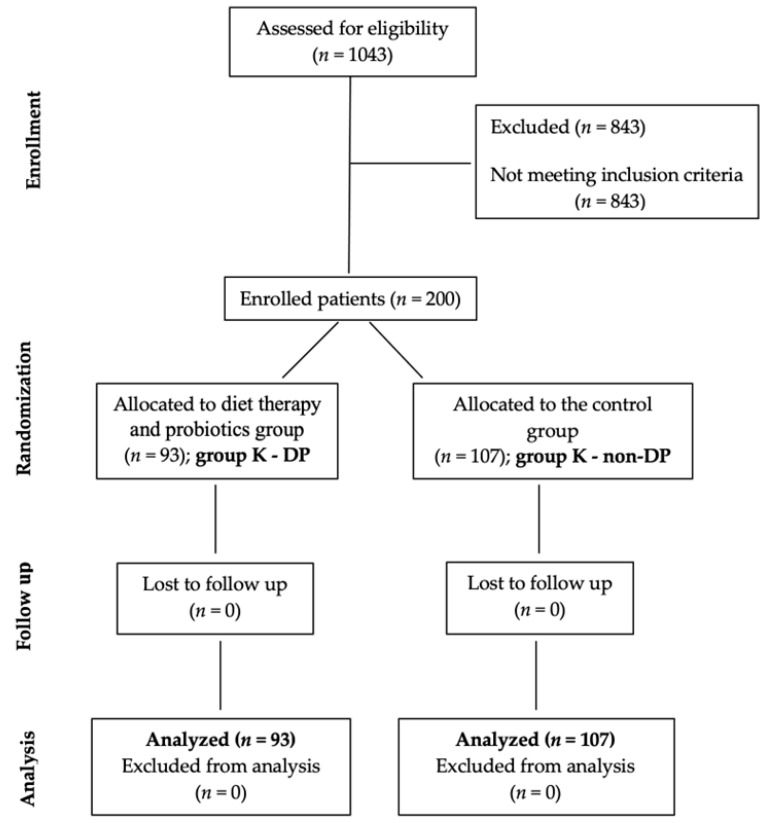
CONSORT flow diagram of the study.

Of the 1043 patients who addressed the center, the Short Physical Performance Battery Protocol and Score Sheet Questionnaire (SPPB) was applied to 382 patients (with a recent history of infection with COVID-19), with the screening role in assessing physical performance. A number of 200 patients with SPPB score < 9 (which raises the probability of sarcopenia) were recruited according to a CONSORT flow chart presented in the Figure 2. The nutritional evaluation was carried out in the private office of Echo Laboratories, by specialized personnel. The selected subjects were randomly distributed into two groups:

- Group K—DP included 93 patients with dietary therapy and probiotics, and
- Group K—non-DP, 107 patients without diet therapy and probiotics.

The recruited patients had a respiratory infection with SARS-CoV-2, having a positive test result 14 days prior to the inclusion. The diagnosis was based on confirmation of COVID-19 infection (positive real-time polymerase chain reaction/rapid immunochromatographic assay). 

All patients received treatment with vitamin D (2000 IU) to improve mitochondrial oxidative phosphorylation function in skeletal muscle [25] and they were included in a specific physical training program lasting 40 min. The program consisted of 10 min stretching and 20 min exercises with progressive resistance (up to 70% of the maximum heart rate, calculated by the formula 220-age in years). The program ended with the return period, lasting 10 min. The identified risk factors for sarcopenia (obesity, smoking, alcohol, and coffee consumption), skeletal muscle index (SMI), physical performance, along with paraclinical parameters (Alb, Hb) were evaluated.

Patients from experimental group (Group K—DP) received additional dietary therapy (protein 1.2–1.5 g/kg, in contrast to the standard requirement of 0.8 g/kg used for the other study groups, distributed over three main meals) and probiotic therapy, for two months. The high-protein diet was recommended by a nutrition specialist and was individualized according to the physiological peculiarities and comorbidities of each patient. For each week, a menu was established with 3 main meals and 2 snacks per day. The sources of protein were meat, dairy, eggs, beans, rice, and foods rich in leucine. To monitor adherence to the diet, the patients were called weekly by the nutritionist and were evaluated monthly from the nutritional point of view.

The probiotic product used in the study was selected based on its composition; it is a standardized nutritional supplement containing 6 bacterial strains (*Enterococcus faecium*, *Lactobacillus acidophilus*, *Lactobacillus brevis*, *Lactococcus lactis*, *Bifidobacterium bifidum*, and *Bifidobacterium lactis*), at least 10 billion organisms per portion = 4 g. The contents of a 4 g sachet are dissolved in 125 mL of water. It is consumed after approximately one minute, the time required for bacterial activation in the morning on an empty stomach or in the evening before going to bed according to the manufacturer’s recommendations (AllergoSan Institute, Austria). Probiotic supplementation was well-tolerated. In the week after administration, a small number of patients (*n* = 8, 8.6%) reported digestive problems (constipation) that improved after regulating water consumption, following the nutritionist’s recommendations.

The study was approved by the institutional review board of the Medical Treatment and Rehabilitation Centre, Băile 1 Mai, Ceres Hotel, Bihor County, Romania (3918/16 November 2020). The research was conducted in compliance with the Declaration of the World Medical Association of Helsinki. Participation in the study was voluntary. Written informed consent was obtained from all participants as a tool for accurate information processing, improved decision-making capacity, collection, and processing of databases.

### 2.2. Study Tools

All patients included in the study were evaluated in terms of body mass index, by relating weight to height. The classification was made as follows: normal weight (18.50–24.99 kg/m^2^), overweight (25.00–29.99 kg/m^2^), first degree obesity (30.00–34.99 kg/m^2^), obesity grade II (35.00–39.99 kg/m^2^), morbid obesity (>40.00 kg/m^2^) [26]. 

**Figure 3:**
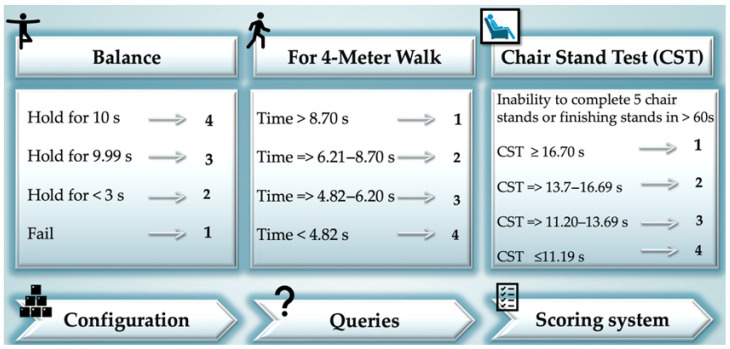
Short Physical Performance Battery Protocol and Score Sheet (SPPB), summarized considering [27].

The Short Physical Performance Battery Protocol and Score Sheet Questionnaire (SPPB) with a screening role was used to evaluate the physical performance of all recruited patients. It considers the ability to rise from a chair, balance, and walking speed [27], as it is described in the Figure 3. Walking speed below 1.0 m/s (4 m distance on flat ground tested), the time to rise from a chair five times (5 CST) extended over 12 s, and a score value ≤ 9 indicate low physical performance. Patients with SPPB score < 9 were evaluated with the Tanita MC-780MA P (Tanita Corporation, Tokyo, Japan) to determine the skeletal muscle index (SMI). Clinical assessment was performed (to determine appendicular muscle mass index (ALM) using a body analyzer. The multifrequency bioelectrical impedance device (MF-BIA) with eight electrodes Tanita MC780MA (Tanita Corporation, Tokyo, Japan) was used. The technique consists of measuring the impedance of the body during the passage of a low-intensity electric current with variable frequencies (5 kHz/50 kHz/250 kHz) [28].

The results were evaluated using the medical software GMON 3.4.1 (Chemnitz, Germany). BIA body analyzers are World Public Health Nutrition Association (WPHNA)-approved devices, used to determine body composition accurately. The margin of error was 0.1 kg. Appendicular skeletal muscle mass (ASM) and skeletal muscle index (SMI) were determined using the formula of Janssen et al. (ASM/height squared) [29]. Published research claims that the MF-BIA method (also used as diagnostic method for sarcopenia in our study) is a useful tool [30], with a specificity of 90%, in evaluating elderly adults [31]. The Asia Working Group on Sarcopenia (2019) suggested that SMI (men, <7.0 kg/m^2^; women, <5.7 kg/m^2^) [32] and sarcopenic obesity were defined as low SMI plus a high percentage of body fat (PBF) (men: low SMI and PBF ≥ 25%; women: low SMI and PBF ≥ 30%) [33,34]. Khanal et al., in their study on sarcopenia, obesity and sarcopenic obesity, established the muscle mass index for healthy people as SMI > 6.67 kg/m^2^ [35], this reference value being used in our study as well.

The training frequency was 3 times a week for 2 months. The initial evaluation was carried out in the first seven days after leaving the quarantine; the final assessment was carried out two months after the first assessment.

Among the biochemical markers were monitored two metabolic parameters (Alb, and Hb) at the beginning and end of the research period. Determining Alb helps monitor the body nutritional status. Serum Alb, the essential protein component of plasma, with a half-life of 21 days, was determined from peripheral venous blood, collected on an empty stomach by the spectrophotometric method (CHEMISTRY VITROS 4600, Ortho Clinical Diagnostics, Inc., Raritan, NJ, USA). Hb, responsible for cellular respiration, was determined by spectrophotometry using the Drabkin method (NIHON Celltac G MEK-9100K-Levant Biotech Company Ltd.—Lusaka, Zambia). The evaluation of the patients was performed at the time of inclusion in the study and after two months.

### 2.3. Statistical Analysis

Statistical analysis was generated using the Statistical Package for the Social Sciences, version 20. Means, standard deviations, and tests of statistical significance were determined. Normal distribution was checked with the Kolmogorov Smirnoff test, the results having a normal distribution (*p* > 0.05). The calculation of the *p*-value was realized using Student t-test for numerical variables, and chi-square test for ordinal variables and frequency. The statistical significance was considered for *p*-values < 0.05. 

The sample size of patients included in the study was calculated for a total number of patients (1043) who addressed the center during the monitored period. The following formula was used:

n = t^2^pq/(Δx^2^ + t^2^pq/N)

where p is the probability of occurrence for the phenomenon, 0 ≤ p ≤ 1; q is against the odds, q = 1 − p; t is the probability factor; Δx is the allowable error limit; and N is the volume of the community. The formula is valid for studies where the observed characteristic is an alternative. For a maximal n, the product pq should be maximal, p = q = 0.5. The probability of 95% corresponds to a value of t = 1.96. The error was set at 0.1. According to our data, the sample size for this study must be at least 96 cases.

## 3. Results

The demographic data, medical history, risk factors for sarcopenia (obesity, smoking, alcohol, and coffee consumption) for the study groups (Group K—DP, Group K—non-DP) are presented in the Table 1. No statistically significant differences between the groups were reported.

The screening for sarcopenia, using the SPPB test revealed a mean value of 4.86 ± 2.57, with 112 (56%) subjects included in the study showing poor physical performance, and 88 (44%) presenting intermediate values (Table 2).

**Figure 4:**
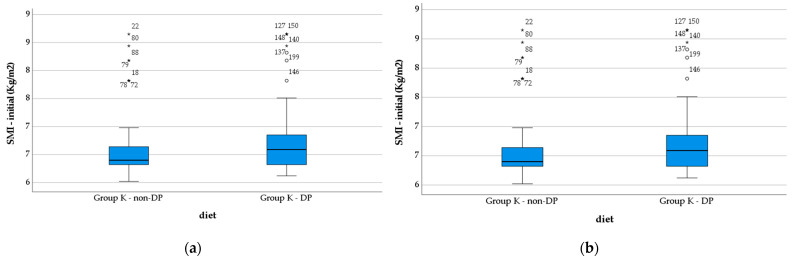
Evolution of the SMI in study groups: (**a**) initial vales; (**b**) final values. Group K—non-DP: Group K without diet therapy and probiotics; Group K—DP: Group K with diet therapy and probiotics. *, extreme outliers; o, mild outliers.

The SMI was initially low for both groups (6.5 ± 0.52 Kg/m^2^ for Group K—non-DP vs. 6.7 ± 0.57 Kg/m^2^ for Group K—DP), without significant statistical differences (*p* = 0.135). The index values raised statistically significantly in Group K—DP compared with Group K—non-DP (6.92 ± 0.50 Kg/m^2^ vs. 6.77 ± 0.56 Kg/m^2^, *p* < 0.048) after 2 months, as it is depicted in the Figure 4.

In Group K—non-DP, no significant statistical modifications were recorded between the number of subjects with sarcopenia (*p* = 0.068) and normal values of SMI (*p* = 0.070), initially and at the end of the intervention (Table 3). In Group K—DP, at the end of the study, compared with the initial moment, there were more patients with normal values of the SMI (*p* < 0.001) and fewer patients with sarcopenia (*p* < 0.001) (Table 3).

**Figure 5:**
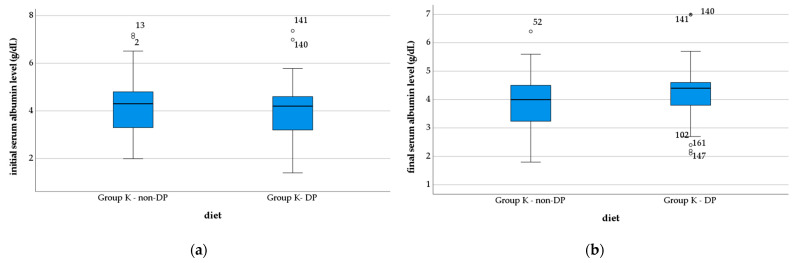
Evolution of the serum albumin level in the study groups: (**a**) initial vales; (**b**) final values. *, extreme outliers; o, mild outliers.

The initial mean values of serum Alb obtained in the two groups (Group K—non-DP, 4.17 ± 1.04 g/dL; Group K—DP, 3.95 ± 0.98 g/dL), were not statistically significantly different (*p* = 0.122). The values became different after the therapeutic intervention, (Group K—non-DP, 3.92 ± 0.82 g/dL; Group K—DP 4.24 ± 0.80 g/dL; *p* = 0.005), according to data presented in the Figure 5.

**Figure 6:**
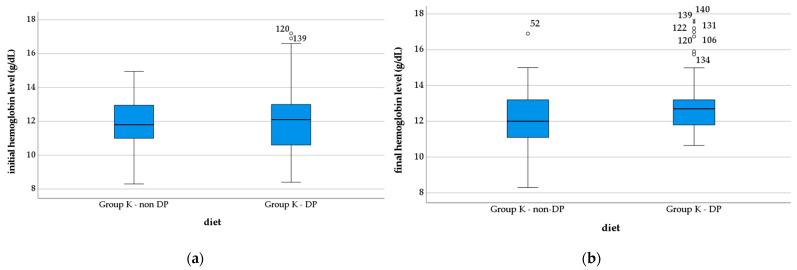
Evolution of the hemoglobin level in the study groups: (**a**) initial vales; (**b**) final values. *, extreme outliers; o, mild outliers.

Hb levels did not statistically significantly differ between the two groups at the time of enrollment in the study (Group K—non-DP, 12.01 ± 1.45 mg/dL; Group K—DP, 12.04 ± 1.81 g/dL; *p* = 0.904). The Hb level improved statistically significantly after the hyper protein diet and probiotics (Group K—non-DP, 12.20 ± 1.54 mg/dL; Group K—DP, 12.83 ± 1.42 g/dL; *p* = 0.003) (Figure 6).

**Figure 7:**
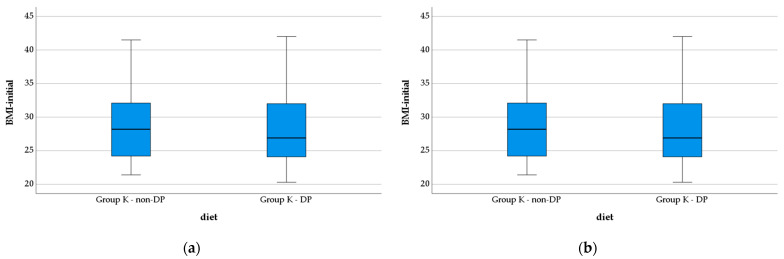
Evolution of the BMI in the study groups: (**a**) initial vales; (**b**) final values.

No statistically significant differences were recorded for IMC values between the two monitored groups either at the time of enrollment in the study (Group K—non-DP, 28.72 ± 4.96; Group K—DP, 28.19 ± 5.22; *p* = 0.464), or at the end of it (Group K—non-DP, 28.72 ± 4.96 mg/dL; Group K—DP, 28.26 ± 5.20 g/dL; *p* = 0.519) (Figure 7).

## 4. Discussion

The accelerated aging of the world’s population is estimated as follows: by 2050, 16% of the world’s population will be over 65 years of age and the number of people over 80 years of age will increase by three times [31]; therefore, the incidence of sarcopenia will also increase. It is considered that more than 50% of people over 80 years of age have sarcopenia [36]. With advancing age, visceral and intramuscular fat accumulates, which is so-called sarcopenic obesity [35]. Screening is essential in clinical practice to intervene with different educational programs related to physical activity or nutrition.

Therapeutic measures to prevent sarcopenia became more accessible after developing detection possibilities (computer tomography and BIA) [20]. Diagnostic methods are constantly being modified; different tests and algorithms have been sought over time to facilitate the identification of subjects with sarcopenia [24,37]. Reduction in the quantity and quality of muscle mass has been correlated with several diseases, such as osteoporosis [27,38] and colorectal cancer [19,39,40], but most often with obesity [41,42]. 

The etiology of sarcopenia is multifactorial; therefore, the therapeutic approach must be complex, including a physical exercise program appropriate to the age, associated pathology, and a diet rich in protein and vitamins [43]. Due to the lack of allopathic treatment for sarcopenia, a diet with additional protein intake combined with probiotics was used in this study to investigate their effects in the evolution of patients with a recent history of COVID-19.

There is increasing evidence that the gut microbiome influences muscle physiology in a number of disorders that cause muscular atrophy [44]. It is proven that the intake of specific probiotics, modifying the intestinal microbiota, favors the increase in skeletal muscle mass [45]. 

The average age of the studied cohort, 67.43 ± 7.94 years, correlates with an increased predisposition to sarcopenia determined by the aging process on the one hand, or by the restriction of activity imposed by the pandemic in various forms (isolation, quarantine, hospitalization) on the other hand. 

The SPPB test was used to highlight sarcopenia with a screening role. SPPB test values < 6 indicate low performance. The average value of the study group was 4.86 ± 2.57, falling into the low-performance range. A total of 44% of the recruited patients obtained initial values that placed them in the intermediate performance range, i.e., values between 6 and 9 of the SPPB test. Additionally, the MF-BIA method was used to determine SMI.

There is a lack of consensus in diagnosis and measurement. Different techniques have been created to assess body structure utilizing various physical concepts, models, and theories. Although dual X-ray absorptiometry (DXA) is frequently used to calculate muscle mass in studies, it is accessible only in a small number of facilities for routine use. When DXA is not accessible, BIA can be used to assess muscle mass in the diagnosis of sarcopenia [46]. The consideration of BIA for assessing sarcopenia is easy and fast. However, the sensitivity of the results depends on the patient’s ethnicity (there are differences between the relevant European and Asian guidelines), medical conditions and comorbidities, hydration, exercise history, and dietary intake [47]. 

The dietary patterns adopted due to isolation and increased sedentary lifestyle has led to the appearance of obesity and sarcopenic obesity [16]. The European Society for Parenteral and Enteral Nutrition (ESPEN) recommends, for the care of patients with SARS-CoV-2, a nutritional supplement with 400 calories and a protein intake of >30 g protein/day, divided into three main meals [48,49,50]. It should be supplemented with 600 kcal daily in people with high risk. Nutritional support must be maintained between 30 and 60 days [51]. In the present study, diet therapy and probiotics were recommended for two months. Moreover, vitamin D intake is essential (daily supplementation of 400 IU), which can have prognostic importance, and the connection between vitamin D deficiency and severe infections is recognized [45]. 

The literature review (2022) emphasizes the need for protein intake of 1.0–1.3 g/kg/day in the elderly to maintain muscle mass and function. This protein intake, higher than the previously published values of 0.8 g/kg/day, will lead to 40% reduction in muscle mass loss. Moreover, the quality of ingested proteins and their distribution in each meal is essential due to the correlation of ingestion with pro-inflammatory factors [2]. These diet therapy recommendations were applied to the experimental group, to ensure the energy needed to restore the body after the infection with COVID-19 and to reduce muscle mass loss [52].

The proteins that reach the intestine are subjected to biochemical transformations. The type of protein is essential; thus, animal proteins are more complex and more efficiently absorbed. 

Supplementation with probiotics is recommended to facilitate the absorption of essential amino acids [24]. The combination of probiotics improves the intestinal microflora homeostasis, reducing fragility and unhealthy aging [53]. Studies on animals (mice) demonstrate the effects of *Lactobacillus plantarum*, with a role in increasing muscle strength and the number of slow-twitch muscles; administration of Bifidobacterium breve B-3 to mice stimulates mitochondrial biogenesis (by increasing phosphorylation and activating the transcriptional coactivator involved in biogenesis, PGC-1α). *Lactobacillus reuteri* modulates the transcription factor N1, with a role in delaying the onset of cachexia in mice with cancer [45]. It was taken into account that the anti-inflammatory properties of *Lactobacilli* might prevent muscular atrophy, which was studied on cachexia mice models [54]. A study on rats indicated that *Enterococcus faecium* supplementation may attenuate the shifts in the typology of whole muscle fibers from slow- to fast-twitch fibers via the upregulation of PGC-1α and the activation of the calcineurin-NFAT signaling pathway, thereby ameliorating the decrease in muscle endurance associated with inactivity [55]. Unfortunately, there are not many clinical studies that look into the influence of the gut flora alteration on sarcopenia. A recent systematic review (published in 2021) analyzed 10 clinical studies, in which 73.5% of the participants were elderly people [44]. None of the research on ageing specifically addressed sarcopenia or properly adhered to the parameters for diagnosing sarcopenia. To separate participants into different groups or to track the effects of therapies, BIA, DXA, physical activity level, and functional tests were recorded. Exercise-induced improvements in function are linked to changes in the gut microbiome, while decreased muscle mass and poor physical function are related to gut microbial dysbiosis [44]. Just three studies provided young people bacterial supplementation [56] as well as to the elderly [57,58]. Depending on several factors (i.e., dose, type, administration interval, and target groups) there is clear disagreement about the benefits of using muscle/sarcopenia supplements. Changes in the gut microbiota influenced the muscle through a variety of mechanisms that partially coincided with the pathophysiology of sarcopenia [44].

In our study, after two months of hyper protein diet associated with probiotics, a significant improvement of SMI was observed. Moreover, at the end of the study the experimental group there were significantly more patients with normal SMI, while in the control group, the number of patients with sarcopenia was insignificantly smaller, despite physical training performed three times a week, 30–40 min each. Regarding the body mass index, no statistically significant differences were recorded between the two monitored groups, at the time of enrollment in the study or at the end of it. Moreover, results of the present study show that after the therapeutic intervention, the average value of Alb and Hb in the group with diet therapy and probiotics increases significantly, indicating an improvement in the general condition of the patients.

The survey carried out by Sanna Vikberg et al. (2019) on 36 patients with sarcopenia, subjected to a 45-min workout (moderate to high intensity on the Borg scale) for ten weeks and a dietary supplement of 175 kcal (21 g protein week 1–7, then 30 g protein week 8–10 of the intervention), supports the significant improvement of the sit-to-stand test by 0.9 ± 0.6 s, compared with the control group, with the overall improvement of the SPPB score. The increase in the sarcopenia score was significant among men. The control group (N = 34) did not show any improvement in the evaluated parameters [59], which underlines the importance of diet therapy (protein intake). Macro- and micronutrients are essential in Hb synthesis, providing energy for the body and maintaining muscle and bone mass. The additional intake of proteins with leucine is important for compensating anabolic resistance and inhibiting muscle degradation [60]. 

The study published by S. Verlaan et al. (N = 417) supports the association of sarcopenia with low protein intake, but nutritional supplements do not have consistent results in sarcopenic patients, the results being influenced by their basal nutritional status [61]. The conclusion of the PROVIDE study (N = 288) is that the intake of 800 IU vitamin D and 20 g of proteins rich in leucine (3 g), for 13 weeks determines the reduction in the chronic inflammatory profile in sarcopenic patients. The modification of pre-albumin occurs with a predictive role in reducing IL-6 [62].

In addition to aging, sarcopenia is associated with malnutrition (often found among elderly people), low physical activity, pro-inflammatory state, and increased oxidative stress. It is important to identify the characteristic biomarkers to prevent or reduce the effects of the decrease in physical performance [63].

Serum Alb is considered a biomarker monitoring the effect of resistance training programs on the elderly [43]. A meta-analysis published in 2022 [6] demonstrates the negative correlation of Alb and Hb with sarcopenia [6]. The correlation of low Alb levels with chronic diseases (morbid obesity [49], diabetes [51], or cancer [2]) is supported. For this reason, the Alb values must be correlated with other parameters. When recruiting patients, patients with a history of malignant tumors or other disabling diseases that limit the patient’s mobility were excluded, for greater accuracy. 

Hb is a well-known biomarker for anemia and nutritional status. The study conducted on 730 patients (2021) showed that a higher Hb level is associated with greater muscle strength and higher walking speed. A significant association between anemia and sarcopenia and a strong correlation between sarcopenia and anemia in men was demonstrated [64]. After administration of the hyper protein diet associated with probiotics, the Hb level improved statistically significantly (*p* = 0.003) for the patients included in the experimental group of this study. The study on stroke patients, published in 2020 [52], demonstrated a strong correlation between low Hb values and sarcopenia. Another study (published in 2020) suggested the correlation of sarcopenia with anemia in men [53]. Low Hb has a complex effect on the body, a risk factor associated with high morbidity and mortality in both inpatients and outpatients [6].

### Limitations and Strengths of the Study

The accuracy of determining sarcopenia with the BIA method is limited due to factors that can influence the assessment (the amount of water or the effort made by the patient), as there are variations within the same day for a patient. Multi-frequency BIA (the method used in this study) has higher accuracy than single-frequency BIA [65]. To limit these shortcomings, the patients were instructed not to consume large amounts of water and not to exert sustained efforts 2–3 h before the measurements [66]. Moreover, the skeletal muscle index values that define sarcopenia are different, depending on the device and the gender of the patient (the limit points for men—6.76 kg/m^2^ and for women—5.28 kg/m^2^, [31]). According to Walowski et al. [67], the values are between 7.70 and 9.20 kg/m^2^ in men and 5.67–7.40 kg/m^2^ in women. The results depend on the hardware and the calculation algorithm.

It must also be considered that protein intake cannot be tracked with great accuracy because the recommendation of protein supplementation is not strictly supervised and the adherence to the physical training program was not monitored. Furthermore, the incidence of diabetes was not evaluated.

The study followed the evolution of sarcopenia and hematological parameters, which characterize nutritional status, in patients with a recent history of infection and prolonged immobilization. It also compared the intervention of diet therapy and probiotics with the evolution of this disorder.

The medical literature claims that SARS-CoV-2 is a virus responsible for the production of dysbiosis, which leads to changes in the immune system, poor absorption of nutrients, and different disorders [68]. Loss of appetite and alteration of taste phenomena present in COVID-19 during the acute period, but also after it, lead to a decrease in the intake of nutrients. The aggravation of these phenomena can be prevented by restoring the normal gut microbiota, adequate intake of nutrients, and restoring muscle mass through physical exercises.

Moreover, COVID-19 is and most likely will remain an infectious disease, with a respiratory route of transmission [69] and a high risk of contamination, thus preserving the need for patient isolation and protection during the acute episode (as for all infectious diseases from this class). Both during the isolation period and during the period immediately following, when post-viral asthenia syndrome and long COVID-19 can set in, patients avoid physical exercises due to the objective impossibility of performing them.

## 5. Conclusions

The prolonged immobilization caused by infection of COVID-19 and the restrictions imposed by the pandemic determine the reduction in physical activity and changes in eating habits with repercussions on the functional state. Diet therapy, consisting of increased protein intake and specific probiotics in muscle anabolism, along with adequate physical training, has demonstrated its role in improving SMI. Significant differences were reported in the improvement of the sarcopenic index values in patients who were recommended an additional intake of proteins and probiotics. More studies to confirm these results and elucidate the mechanism through which some probiotics can influence sarcopenia are necessary. Understanding how specific dietary interventions associated with probiotics can prevent muscle loss can help improve the clinical and therapeutic management of infected patients in order to reduce the health consequences of COVID-19.

## Figures and Tables

**Table 1:** Baseline characteristics of the groups.

Parameter	Group K—DP	Group K—Non-DP	*p*
N	93	107	0.322 **
Age, M, SD	68.03 ± 8.09	66.91 ± 7.81	0.503 *
Female, N (%)	66 (33.0)	80 (40.0)	0.233 **
Rural area, N (%)	47 (23.5)	49 (24.5)	0.430 **
BMI, M, SD	28.72 ± 4.97	28.19 ± 5.93	0.850 *
Smoker, N (%)	19 (9.5)	25 (12.5)	0.319 **
Alcohol user, N (%)	8 (4)	12 (6)	0.220 **
Coffee user, N (%)	25 (12.5)	27 (13.5)	0.599 **
Kyphosis, N (%)	90 (45)	102 (51)	0.299 **
Scoliosis, N (%)	64 (32)	68 (34)	0.118 **

Legend: M: mean value; SD: standard deviation value; N: number of the patients; BMI: body mass index; Group K—non-DP: Group K without diet therapy and probiotics; Group K—DP: Group K with diet therapy and probiotics; *p* values statistical significance (*, *t*-test; **, chi-square test).

**Table 2:** Results of the SPPB test applied for sarcopenia screening.

Parameter	Scoring Points	N (%)
Balance	0	8 (4)
	1	40 (20)
	2	152 (76)
For 4-m walk	0	4 (2)
	1	60 (30)
	2	84 (42)
	3	40 (20)
	4	12 (6)
Chair standing Test	0	24 (12)
	1	48 (24)
	2	32 (16)
	3	24 (12)
	4	72 (36)
Score value, M, SD	4.86 ± 2.57	

**Table 3:** Grouping of subjects according to the SMI.

Patients		N (%)		*p*
		Initial	Final	
Group K—non-DP	sarcopenia	70 (75.26)	56 (52.33)	0.068
	normal value	23 (24.73)	37 (47.66)	0.070
Group K—DP	sarcopenia	75 (80.64)	37 (34.57)	<0.001
	normal value	32 (19.35)	70 (65.42)	<0.001

## Data Availability

Data of the patients are available in the medical archive of the Echo Laboratories.
